# Effects of calcium lactate on *in vitro* fertilization and embryonic development in cattle

**DOI:** 10.5713/ab.24.0636

**Published:** 2024-11-06

**Authors:** Bo-myeong Kim, Song-Hee Lee, Geun Heo, Ji-Dam Kim, Gyu-Hyun Lee, Jae-Min Sim, Kwang Taek Lim, Xiang-Shun Cui

**Affiliations:** 1 Department of Animal Science, Chungbuk National University, Cheongju, Korea; 2 Genetics, Anseong, Korea

**Keywords:** Calcium Lactate, Cattle, Embryonic Development, *In Vitro* Fertilization, Oxidative Stress, TALP Medium

## Abstract

**Objective:**

Growing demand for embryo transfer is steadily expanding and further studies on in vitro fertilization of cattle. To assess the effect of calcium lactate by replacing Tyrode’s albumin lactate pyruvate (TALP) medium composition during fertilization and embryonic development.

**Methods:**

Sodium lactate and CaCl_2_ were replaced with 2.0, 3.0, 4.5 mM calcium lactate for TALP medium during fertilization in Experiment 1. In Experiment 2, the concentrations of sodium lactate and CaCl_2_ were re-modified as control, in comparison with the same concentration of calcium lactate at 4.5 mM. Zygotes were moved to sequential media to match early-and late-stage environments. Embryonic development was examined on day 8 after insemination.

**Results:**

A 4.5 mM calcium lactate enhanced the rate of fertilization and blastocyst formation (p<0.0001, p<0.01, respectively). It represented differences in the reactive oxygen species (ROS) (p<0.01) and glutathione (GSH) levels (p<0.05) and increased blastocyst diameter and total cell number (p<0.05). In Experiment 2, fertilization (p<0.05) and blastocyst formation rates (p<0.01) were increased in 4.5mM calcium lactate under same concentration effect of sodium lactate and CaCl2. Additionally, it reduced the ROS (p<0.01) and increased the GSH levels (p<0.05), leading increase embryo quality.

**Conclusion:**

The replacement of calcium lactate in TALP medium enhances fertilization and embryonic development while also improving oxidative stress. Specifically, it has been determined that a concentration of 4.5 mM calcium lactate is the most effective, irrespective of the varying concentrations of sodium lactate and CaCl_2_. This study presents a novel formulation of a modified TALP medium intended for implantation withing the bovine embryo industry. The current implications of the study are discussed in relation to previously stated objectives and hypotheses.

## INTRODUCTION

Assisted reproductive technologies (ART) are extensively employed in diverse fields, including the realm of dairy farming. Particularly, ART techniques utilized in dairy farming encompass artificial insemination with conventional or X-sorted semen, embryo transfer subsequent to multiple ovulations, and *in vitro* fertilization (IVF) [1]. The *in vitro* production (IVP) of bovine embryos has attained a prominent position in the dairy genetic improvement field over the past decade [2]. However, bovine IVF embryos exhibited a diminished developmental potential, which was partially attributed to modifications in the epigenetic profile of the gametes, necessitating further enhancement in research [3]. Similarly, research on media for embryo culture has continued over the past few decades [4]. However, given that embryos are used in diverse industries, there is a constant need to enhance their quality.

In cattle, a critical component of the IVP process involves the fertilization of mature oocytes using high-quality bull sperm [5]. Oocyte activation, an essential series of events that take place prior to embryogenesis during fertilization, is carefully regulated by specific patterns of intracellular calcium (Ca^2+^) release observed across various species [6]. Ca^2+^ oscillations are initiated by the sperm-specific phospholipase C zeta (PLCζ). Earlier investigation has demonstrated that sperm cells lacking normal PLCζ function, as achieved through RNA interference in mince, exhibit only irregular Ca^2+^ oscillations [7]. Consequently, these mice experience reduced litter sizes [7], and embryos produced by sperm from PLCζ knock-out mice display delayed developmental profile. These findings suggest a decline in both sperm and epididymal quality [8]. The release of Ca^2+^ driven by PLCζ may therefore play a crucial role in regulating the rate of cell cycle progression, thereby influencing the efficiency of embryonic development [9].

The most common fertilization medium used for IVF is Tyrode’s albumin lactate pyruvate (TALP). Calcium is considered as one of key factors for sperm capacitation. Chelating agents that can be used in embryo culture include ethylene-diamine-tetraacetic acid and organic acids such as lactic acid, citric acid, and acetic acid. Calcium lactate is considered as one of the chelated forms. Wherein calcium is bound to an organic acid. It is synthesized through the combination of lactic acid with either calcium carbonate or calcium hydroxide. In commercial culture media, there are single medium and sequential medium in which calcium lactate is used [10]. However, its content has not been accurately determined for fertilization media. Bovine sperm have a highly active calcium ATPase in their plasma membrane, which helps expel calcium and maintain intracellular calcium (Ca_i_) within the nanomolar range [11]. An essential final increase in Ca_i_ is associated with physiological capacity of the acrosome reaction [12].

Although studies on various fertilization media have been reported [13,14], studies on the calcium lactate concentration of TALP media, which is the most effective in cattle, have not been conducted. Therefore, the purpose of this study is to investigate whether it is efficient to use calcium lactate instead of CaCl_2_ and sodium lactate in TALP media and to evaluate at what concentration it is most effective.

## MATERIALS AND METHODS

All animal studies were approved and performed according to the guidelines of the Institutional Animal Care and Use Committee (IACUC) of Chungbuk National University, Korea. Ovaries were used from the slaughtered cows, which does not include animal studies.

### Experimental design

#### Experiment 1

To evaluate the impact of calcium lactate on TALP medium, as opposed to the combination of sodium lactate and CaCl_2_, mature oocytes were fertilized with sperm in modified TALP medium containing 2.0, 3.0, and 4.5 mM of calcium lactate, respectively. The components of the control and treatment groups are displayed in Table 1. The total number of cumulus-oocyte complexes (COCs) in the control group was 1,177, and the treatment groups at varying concentrations were 1,057, 1,130, and 1,143, respectively. Subsequently, the rate of fertilization and blastocyst formation were examined to identify the most effective concentration of calcium lactate. Oxidative stress levels and embryo quality were assessed.

#### Experiment 2

The concentration of sodium lactate and CaCl_2_ was adjusted to match the control group, in comparison to the same concentration of calcium lactate used in Experiment 1, which proved to be the most effective alternative for investigating the replacement effect of calcium lactate in each compound. The compositions are presented in Table 2. The experimental evaluation was conducted in the same manner as in Experiment 1.

### Collection of oocyte and *in vitro* maturation (IVM)

Ovaries of Han-woo cows were obtained from a local slaughterhouse (Dodram LPC, Anseong, South Korea) within two hours after slaughtering and transported to the laboratory in a thermos bottle containing 37°C normal saline. COCs from follicles with a diameter of 2 to 8 mm was aspirated using a 19-gauge needle connected to a 10-mL single-use syringe [15]. COCs with three or more layers were selected and washed thrice in 25 mM 4-(2-hydroxyethyl)-1-piperazineethanesulfonic acid (HEPES)-buffered medium-199 (Medium-199; Gibco, Waltham, MA, USA) supplemented with 0.1% bovine serum albumin (BSA), 10 mM NaHCO_3_ (Sigma-Aldrich, St. Louis, MO, USA), and 1% penicillin-streptomycin. For maturation, approximately 10 to 15 COCs were maturated for 22 hours in each 50 μL Medium199 micro drops overlaid with paraffin liquid (Daejung, Siheung, Korea.) and cultured in a humidified incubator at 38.5°C 5% CO_2_. Maturation medium was supplemented with 0.1% BSA, 0.005 IU/mL follicular stimulating hormone (Antrin, Teikoku, Japan), and 1 μg/mL 17-β estradiol (Sigma-Aldrich) [15].

### *In vitro* fertilization

After IVM, oocytes were washed thrice in a HEPES-buffered TALP washing medium. Frozen-thawed semen of a bull (Hanwoo Genetic Improvement Center, Seosan, Korea) was subjected to Percoll gradient centrifugation to separate the motile sperm [16] and then inseminated with 1×10^6^ spermatozoa/mL for 18 hours in 10 oocytes per 43 μL drops of TALP-IVF medium (pH 7.4, 290 mOsm/kg; Table 1, 2) in a humidified incubator at 38.5°C 5% CO_2_ [15].

### In vitro culture

Twenty hours after insemination, a glass pipette was used to remove the cumulus cells and attached sperm surrounding the zygotes. Sequential media [15] was used to match early- and late-stage environments. Approximately, 15 to 20 zygotes were cultured per 20 μL micro drops of early-stage medium containing NaCl 107.70 mM, KCl 7.16 mM, NaHCO_3_ 25.07 mM, KH_2_PO_4_ 1.19 mM, Sodium-lactate 6.60 mM, Na-pyruvate 0.33 mM, CaCl_2_ 1.71 mM, MgCl_2_ 0.49 mM, HEPES 5 mM, Glucose 1.50 mM, non-essential amino acids (NEAA) 1%, and polyvinyl alcohol (PVA) 0.1 mg/mL at 39°C in a humidified incubator of 5% O_2_, 5% CO_2_ and 90% N_2_. After 120 h in early stage medium, embryos were washed twice in later-stage medium and cultured for 96 h per 20 μL microdrops of later-stage medium containing NaCl 107.70 mM, KCl 7.16 mM, NaHCO_3_ 25.07 mM, KH_2_PO_4_ 1.19 mM, sodium-lactate 3.30 mM, Na-pyruvate 0.11 mM, CaCl_2_ 1.71 mM, MgCl_2_ 0.49 mM, HEPES 5 mM, glucose 1.50 mM, EAA 2%, NEAA 1%, and PVA 0.1 mg/mL (pH 7.4, 290 mOsm/kg) at 39°C in a humidified incubator with 5% O_2_, 5% CO_2_, and 90% N_2_. The fertilization rates were examined on day two after insemination. The rate of blastocysts formation was examined on day eight after insemination confirming the stage.

### Measurement of reactive oxygen species and glutathione

Total reactive oxygen species (ROS) levels were measured in blastocysts on day eight using 2’,7’-dichlorodihydrofluorescein diacetate ([H2DCF-DA] Cat#D399; Molecular Probes, Eugene, OR, USA) [17]. In summary, blastocysts were cultured for 15 min at 38.5°C in PVA-phosphate-buffered saline (PBS) with 10 μM H2DCF-DA. To explore glutathione (GSH) levels, blastocysts that were washed thrice with PBS/PVA were incubated in PBS/PVA containing 10 μM Thiol-Tracker Violet (Cat #T10095; Thermo Fisher Scientific, Waltham, MA, USA) at 38.5°C for 30 min and then washed thrice with PBS/PVA. Fluorescent signals were captured using a digital camera (DP72; Olympus, Tokyo, Japan) attached to a fluorescence microscope (IX70; Olympus), as described previously. By utilizing the Fiji ImageJ software (National Institutes of Health), the fluorescence intensity of blastocysts was analyzed to estimate ROS and GSH levels. The fluorescence intensity was arbitrarily set to 1 in the control group and the relative intensity in the treatment group was determined.

### Measurement of total cell number

The total number of cells in blastocysts was measured on day eight. After washing thrice with PBS/PVA, the blastocysts were fixed in a 3.7% formaldehyde solution at 25°C for 30 min in a 96-well plate. After 30 min, the samples were washed thrice with PBS/PVA to formaldehyde. Blastocysts were permeabilized with PBS/PVA containing 0.5% Triton X100 at 25°C for 30 min in 72-well plates (20μL per well). After washing thrice with PBS/PVA, the blastocysts were mounted on glass slides using a 4′,6-diamidino-2-phenylindole solution. A digital camera (DP72; Olympus) attached to a fluorescence microscope (IX70; Olympus) was used to capture the fluorescence signals and images were saved as .jpg files. The number of cells within the blastocyst was counted using Fiji ImageJ software (National Institutes of Health).

### Real-time reverse transcription-quantitative polymerase chain reaction

All procedures employed the previously described method for real-time reverse transcription-quantitative polymerase chain reaction (RT-qPCR) [17,18]. Briefly, mRNA was extracted from 20 to 40 blastocysts. The mRNA from each group was extracted using the DynaBeads mRNA Direct kit (61012; Thermo Fisher Scientific) according to the manufacturer’s guidelines. Using SuperScript3 Reverse Transcriptase (Thermo Fisher Scientific) and Oligo (dT) 20 primers, the mRNA was reverse-transcribed to generate cDNA. A WizPure qPCR Master kit (WizBiosolutions, Loco Hills, NM, USA) was used to perform real-time qPCR. Reaction mixtures with a final volume of 20 μL contained 10 μL of SYBR Green, 1 μL of each of the forward and reverse primers, 2 μL of cDNA template, and nuclease free water. The amplification conditions were as follows: initial denaturation at 95°C for 10 min, followed by 40 cycles of amplification at 95°C for 15 s, 60°C for 20 s, and 72°C for 15 s, and a final extension at 95°C for 15 s. The target genes *GPX4*, *IGF2R*, and *GAPDH* were used as reference genes to explore the activity of antioxidant and growth factor, respectively. The primers used to amplify the genes are listed in Table 3. The mRNA expression analysis was performed using the 2^−ΔΔCt^ method, and Ct values from each sample were normalized to housekeeping gene expression and then calculated to the control group as relative expression. All the samples were technically repeated at least three times. To test primer specificity, melting curve, final step to dissolve the double strand of DNA, was confirmed as single peak.

### Statistical analysis

The results were analyzed using GraphPad Prism 5 software (GraphPad, San Diego, CA, USA). All data were subjected using the Student’s t-test and presented as means±standard error of the mean. Statistical significance was set at p<0.05. Each experiment was conducted with at least three independent replicates.

## RESULTS

### Experiment 1

#### Calcium lactate at a concentration of 4.5 mM increases the rate of fertilization and blastocyst formation

**Figure 1:**
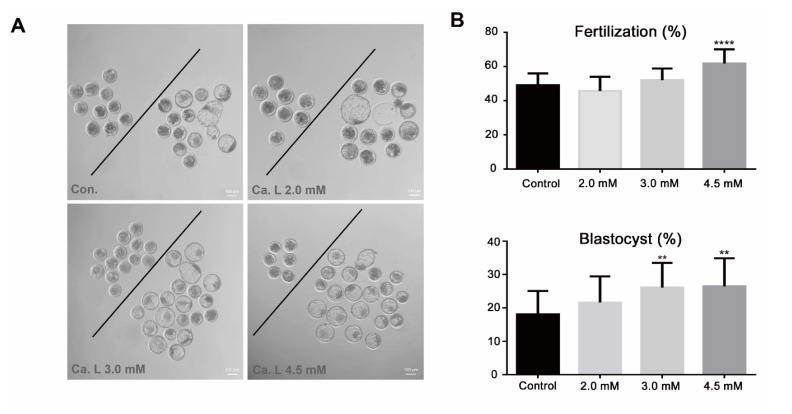
Fertilization and blastocyst formation rates in day eight blastocysts. (A) Morphology of embryos after eight days of fertilization. The scale bar represents 100 μm. (B) Rate of fertilization and blastocyst formation (%) in Control under different concentrations of calcium lactate. Means±standard error of the mean; ** p<0.01, **** p<0.0001.

To determine whether calcium lactate affects fertilization and blastocyst formation, oocytes were cultured in an IVF medium supplemented with calcium lactate (2, 3, and 4.5 mM) for 18 h. The fertilization rate was significantly increased in the 4.5mM calcium lactate group compared to the control group (Figure 1; p<0.0001). Additionally, blastocyst formation was significantly increased in the calcium lactate groups at concentrations of 3 mM and 4.5 mM compared to that in the control group (Figures 1A, 1B; p<0.01). Therefore, 4.5 mM calcium lactate was used for subsequent experiments. These results suggest that 4.5 mM calcium lactate enhances fertilization and blastocyst formation in bovine embryos.

#### Calcium lactate at a concentration of 4.5 mM reduces oxidative stress

**Figure 2:**
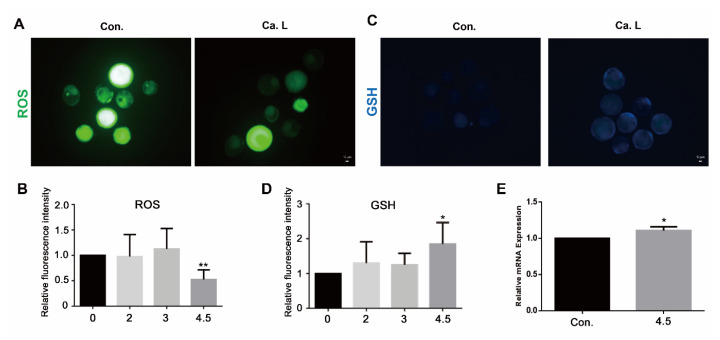
Oxidative stress in day eight blastocyst. (A and B) Images of reactive oxygen species (ROS) levels in CON (n = 32) and different concentrations of calcium lactate (2, 3, and 4.5 mM) (n = 32, 31, 30). The scale bar represents 10 μm. (C and D) Images of glutathione (GSH) levels in CON (n = 48) and different concentrations of calcium lactate (2, 3, and 4.5 mM) (n = 47, 42, 44). The scale bar represents 10 μm. (E) Relative mRNA expression of GPX4 associated antioxidant. All data represents as means±standard error of the mean. * p<0.05 and ** p<0.01 represent significant differences between Con and 4.5 mM calcium lactate. Con, control; Ca. L, calcium lactate.

The fluorescence intensities of ROS and GSH were measured to evaluate oxidative stress and antioxidant potential. As shown in Figures 2A, 2B, the relative intensity of ROS was significantly reduced in the 4.5 mM calcium lactate group (p<0.01), while the relative intensity of GSH (Figures 2C, 2D) was significantly enhanced (p<0.05). In addition, the RT-qPCR results indicated in Figure 2E show that the mRNA expression of *GPX4* (p<0.05) significantly increased with the 4.5 mM calcium lactate treatment, indicating its potential to resist oxidative stress.

#### Calcium lactate at a concentration of 4.5 mM enhances embryo quality

**Figure 3:**
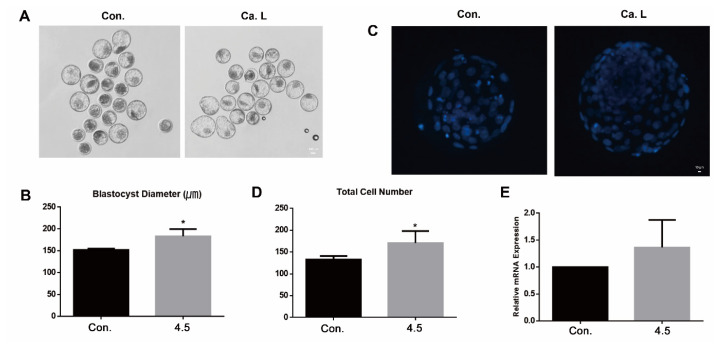
Evaluation of the embryo quality. (A and B) Blastocyst diameter (μm) at day eight. The scale bar represents 100 μm. (C and D) Total number of blastocysts at day eight. The scale bar represents 10 μm. (E) Relative mRNA expression of *IGF2R* associated growth factor. All data represents as means±standard error of the mean. * p<0.05 indicates significant differences between CON and 4.5 mM calcium lactate. CON, control; Ca. L, calcium lactate.

To assess embryo quality following calcium lactate treatment, diameter and total cell number of blastocyst on day 8 were investigated. In present results, the blastocyst diameter was significantly higher at 4.5 mM calcium lactate (Figures 3A, 3B; p<0.05). Additionally, the total cell number of blastocysts was significantly elevated in the treated group (Figures 3C, 3D; p<0.05). Relative mRNA expression of *IGF2R* showed no significant difference between the control and the calcium lactate group (Figure 3E). Collectively, calcium lactate possesses the potential to enhance the quality of embryos during IVP in cattle.

### Experiment 2

#### Calcium lactate at a concentration of 4.5 mM increases the rate of fertilization and blastocysts

**Figure 4:**
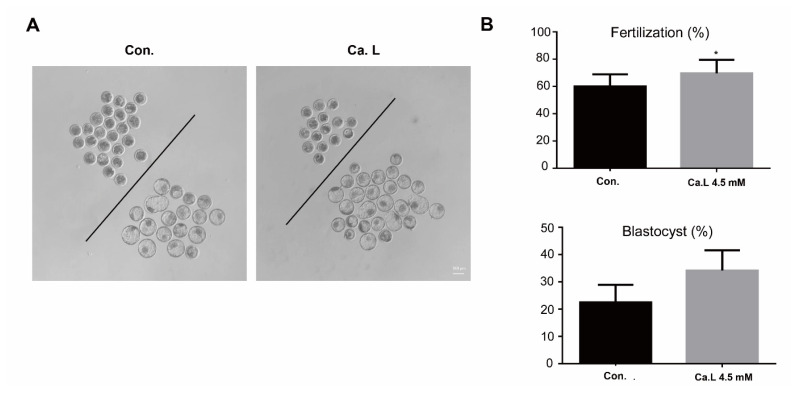
Rate of fertilization and blastocyst formation in day eight blastocysts. (A) Blastocysts at day eight. The scale bar represents 100 μm. (B) Fertilization and blastocyst formation rates (%) in CON and 4.5 mM calcium lactate. Means±standard error of the mean; * p<0.05, ** p<0.01. CON, control; Ca. L, calcium lactate.

Experiment 2 involved conducting measurements of the identical processes as those performed in the preceding experiment. However, in Experiment 2, the concentration of sodium lactate and CaCl_2_ were adjusted to serve as alternative control factors in comparison to calcium lactate at a concentration of 4.5 mM, as employed in Experiment 1. In present results, calcium lactate at a concentration of 4.5 mM caused a significant increase in the rate of fertilization (control: 60±2.81% vs. ca.L 4.5 mM: 70±3.12%; p<0.05) and blastocyst formation (control: 23±2.74% vs. ca.L 4.5 mM: 34±2.33%; p<0.01), in comparison with the same concentration of sodium lactate and CaCl_2_ at 4.5 mM as re-modified control. These results suggest that a concentration of 4.5 mM calcium lactate is the most effective, irrespective of the varying concentrations of sodium lactate and CaCl_2_ (Figure 4).

#### Calcium lactate at a concentration of 4.5 mM reduces oxidative stress

**Figure 5:**
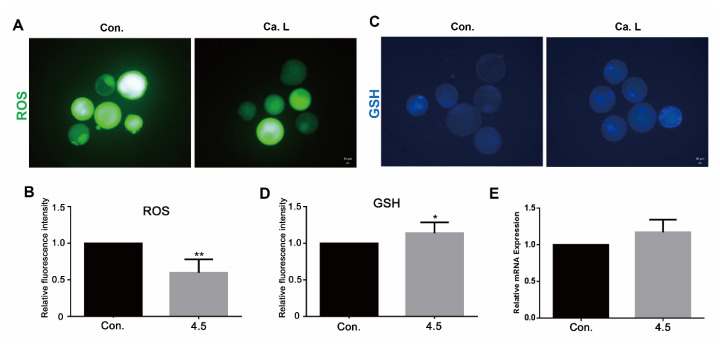
Oxidative stress in day eight blastocysts. (A and B) Images of ROS levels in CON (n=26) and 4.5 mM calcium lactate (n = 26) groups. The scale bar represents 10 μm. (C and D) Images of GSH levels in CON (n = 45) and 4.5 mM calcium lactate (n=44) groups. The scale bar represents 10 μm. (E) Relative mRNA expression of *GPX4* associated antioxidant. * p<0.05, ** p<0.01 indicate significant differences between CON and calcium lactate. All data represents as means±standard error of the mean. CON, control; Ca. L, calcium lactate; ROS, reactive oxygen species; GSH, glutathione.

In further investigation on oxidative stress, the relative intensity of ROS level was significantly reduced in the 4.5 mM calcium lactate group (Figure 5A, 5B; p<0.01). On the other hand, the GSH level of the 4.5 mM calcium lactate group was significantly elevated (Figures 5C, 5D; p<0.05). As shown in Figure 5E, the relative mRNA expression of *GPX4* was not significantly different between the control and treated groups. In brief, calcium lactate has been found to effectively reduce oxidative stress, independent of the concentration of the administered dose.

#### Calcium lactate at a concentration of 4.5 mM enhances embryo quality

**Figure 6:**
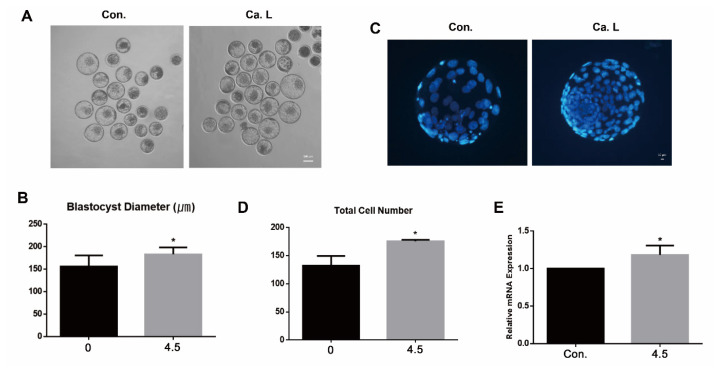
Evaluation of the embryo quality. (A and B) Blastocyst diameter (μm) at day eight. The scale bar represents 100 μm. (C and D) Total number of blastocysts at day eight. The scale bar represents 10 μm. (E) Relative mRNA expression of *IGF2R* associated growth factor. * p<0.05 indicates significant differences between CON and 4.5 mM calcium lactate. All data represents as means±standard error of the mean. CON, control; Ca. L, calcium lactate.

Similarly, the quality of the embryo was confirmed by measuring the blastocyst diameter (Figures 6A, 6B; p<0.05) and total cell number (Figures 6C, 6D; p<0.05). Both experiments showed a significant increase in the 4.5 mM calcium lactate group. In addition, the relative mRNA expression of *IGF2R* was significantly upregulated in the presence of 4.5 mM calcium lactate (Figure 6E; p<0.05). In conclusion, the utilization of calcium lactate has been shown to enhance the quality of embryos during bovine IVF embryos.

## DISCUSSION

The primary objective of our study is to determine the ideal concentration of calcium lactate for modifying TALP medium to enhance cow IVF. Based on our findings, a concentration of 4.5 mM of calcium lactate instead of calcium chloride and sodium lactate proved to be the most effective in achieving successful fertilization and promoting subsequent embryonic development.

In a previous study, calcium concentration in oviduct fluid was estimated to be 4.58±0.324 mM [19]. The ionic composition of the oviduct is physiologically significant, as it affects sperm motility, function, and capacitation [19]. Calcium also plays a critical role in sperm viability, sperm-zona pellucida binding, and acrosome reactions [14,20]. In our first experiment, using calcium lactate at a concentration more than twice as high as the conventional 2.0 mM, the fertilization rate was increased, suggesting that an increase in calcium concentration facilitated the capacitation of spermatozoa. In addition, the increased fertilization rate indicates the potential of calcium lactate to facilitate increased blastocyst formation. The frequency and amplitude of Ca^2+^ oscillations have been shown to impact protein profiles in early embryos, embryonic compaction, blastocyst formation, and the rate of successful transplantation of 4-cell embryos in female mice and rabbits [21]. Furthermore, these oscillations are directly implicated in cell cycle progression, as varying Ca^2+^ transients result in different rates of cell cycle progression [22]. In present study, the rate of blastocyst formation was significantly enhanced in both 3.0 mM and 4.5 mM of calcium lactate groups, suggesting 4.5 mM calcium lactate positively affects fertilization and embryonic development.

In the second experiment, the concentrations of sodium lactate and CaCl_2_ were modified as control, comparing with same concentration of calcium lactate as Experiment 1 to investigate the replaced effect of calcium lactate from each compound. Although TALP media was re-modified alternating composition of sodium lactate and CaCl_2_, 4.5 mM calcium lactate has been shown an increase of fertilization rate and further embryo development. In a previous *in vivo* study, chloride and sodium were the primary ions in oviduct fluid [19]. Calcium lactate, when subjected to modification in TALP medium, functions as an organic chelating agent. The chelated calcium derived from this process does not bind to other metal ions, thereby enhancing its efficacy in the medium. Indeed, the PLCζ, may influence the efficacy of embryogenesis via profiles of elicited Ca^2+^ release [9], so that calcium lactate might influence the oocyte activation, as Ca^2+^ oscillation, inducing those sperm function. This modification may potentially have a positive impact on fertilization rates and the overall quality of embryos.

ROS and GSH levels were measured to confirm oxidative stress. ROS play a significant dual role in capacitation of spermatozoa. At physiological levels, ROS are responsible for regulating a variety of cellular processes, such as the elevation of cyclic adenosine monophosphate, calcium levels, and activation of phosphorylation events that are essential for the process of capacitation [23]. Higher concentrations of ROS promote the oxidation of lipids, proteins, and DNA, ultimately resulting in demise of cells. These detrimental effects have been closely linked to male infertility [23]. Especially, ROS are significant elements that may negatively affect the results of assisted reproductive techniques [24]. However, calcium polypeptide has been shown its effect on increasing peroxidase activity during environmental stress response [25]. Our study showed that calcium lactate led to a significant reduction in oxidative stress, consequently resulting in a notable increase in the fertilization rate. Previous studies have indicated that reducing oxidative stress leads to improved rates of fertilization and embryonic development [26].

Embryo quality is an essential evaluation tool for ART and other industries involving embryos. Embryo quality is evaluated in terms of blastocyst diameter and total cell number. In both experiments, we confirmed that utilization of calcium lactate increased blastocyst diameter, as well as total cell number [17,27]. Collectively, these results indicate that using calcium lactate, results in an increase in embryo quality and would suggest that many animals may stand to benefit from various therapeutic or applications in methods that are relevant to their needs.

In conclusion, the addition of calcium lactate increased the fertilization rate and embryonic formation. It improved the embryonic quality in the IVF of cattle and ultimately enabled the transfer of high-quality embryos. This study contributes to effective embryo production and quality improvement in the cattle IVP industry.

## Figures and Tables

**Table 1:** Composition of the fertilization-TALP medium in Experiment 1

Substrates	Control	T1 Ca.L 2 mM	T2 Ca.L 3 mM	T3 Ca.L 4.5 mM
NaCl	114 mM	114 mM	114 mM	114 mM
KCl	3.2 mM	3.2 mM	3.2 mM	3.2 mM
NaH_2_PO_4_·2H_2_O	0.4 mM	0.4 mM	0.4 mM	0.4 mM
MgCl_2_	0.5 mM	0.5 mM	0.5 mM	0.5 mM
CaCl_2_	2 mM	-	-	-
NaHCO_3_	25 mM	25 mM	25 mM	25 mM
Na pyruvate	0.5 mM	0.5 mM	0.5 mM	0.5 mM
Na-lactate	10 mM	-	-	-
Ca-lactate	-	2 mM	3 mM	4.5 mM
Glucose	1.5 mM	1.5 mM	1.5 mM	1.5 mM
Phenol red	0.005 g/L	0.005 g/L	0.005 g/L	0.005 g/L
BSA	8 g/L	8 g/L	8 g/L	8 g/L
EAA^1)^	2%	2%	2%	2%
NEAA^2)^	1%	1%	1%	1%

1) MEM essential amino acids (11130-051; Gibco, Waltham, MA, USA).

2) MEM non-essential amino acids (11140-050; Gibco).

TALP, Tyrode’s albumin lactate pyruvate; BSA, bovine serum albumin.

**Table 2:** Composition of the fertilization-TALP medium in Experiment 2

Substrates	Control Cacl_2_ 4.5 mM Na lactate 9 mM	T Calcium lactate 4.5 mM
NaCl	114 mM	114 mM
KCl	3.2 mM	3.2 mM
NaH_2_PO_4_·2H_2_O	0.4 mM	0.4 mM
MgCl_2_	0.5 mM	0.5 mM
CaCl_2_	4.5 mM	-
NaHCO_3_	25 mM	25 mM
Na-pyruvate	0.5 mM	0.5 mM
Na-lactate	9 mM	-
Ca-lactate	-	4.5 mM
Glucose	1.5 mM	1.5 mM
Phenol red	0.005 g/L	0.005 g/L
BSA	8 g/L	8 g/L
EAA^1)^	2%	2%
NEAA^2)^	1%	1%

1) MEM essential amino acids (11130-051; Gibco, Waltham, MA, USA).

2) MEM non-essential amino acids (11140-050; Gibco).

TALP, Tyrode’s albumin lactate pyruvate; BSA, bovine serum albumin.

**Table 3:** Primer sequences used in the RT-qPCR

Gene	Primer sequences	Accession
*GAPDH*	F: 5’-CATCACCATCTTCCAGGAGCGAGA R: 3’-CCTGCTTCACCACCTTCTTGATGT	NM_001034034.2
*GPX4*	F: 5’-TGTGCTCGCTCCATGCACGA R: 3’-CCTGGCTCCTGCCTCCCA	NM_001346430.1
*IGF2R*	F: 5’-CGCCTACAGCGAGAAGGGGTTAGTC R: 3’-AGAAAAGCGTGCACGTGCGCTTGTC	NM_174352.2

RT-qPCR, real-time reverse transcription-quantitative polymerase chain reaction; *GAPDH*, glyceraldehyde 3-phosphate dehydrogenase; F, forward primer; R, reverse primer; *GPX4*, glutathione peroxidase 4; *IGF2R*, insulin like growth factor 2 receptor.
